# Designing and evaluating a health education session on respiratory infections addressed to caregivers of children under three years of age attending day-care centres in Porto, Portugal: A community-based intervention

**DOI:** 10.1080/13814788.2016.1240777

**Published:** 2017-03-08

**Authors:** Ana Manuela Ferreira da Silva Alexandrino, Rita Isabel Garrido Vieira dos Santos, Maria Cristina Damas Argel de Melo, José Adelino Mesquita Bastos

**Affiliations:** ^a^ Department of Physiotherapy, School of Allied Health Technologies, Polytechnic Institute of PortoPortugal; ^b^ Department of Health Sciences, University of AveiroPortugal; ^c^ Faculty of Sports, University of PortoPortugal; ^d^ Baixo Vouga Hospital CentreAveiroPortugal

**Keywords:** Health education, public health and community medicine, patient involvement empowerment, self-management, paediatrics, infectious diseases, prevention

## Abstract

**Background:** Acute respiratory infections (ARI) are common in children, increasing the pressure on clinicians to prescribe antibiotics and affecting public health

**Objectives:** This study aimed to design a health education session (HES) for caregivers of children, and to evaluate its effects on caregivers’ needs, as well as on their knowledge and attitudes concerning ARI.

**Methods:** A generalized model of developing, implementing and evaluating a community-based intervention was followed, including caregivers of children under three years of age. Caregivers were randomly distributed into an intervention group (IG) (*n* = 41) and a control group (CG) (*n* = 51) and the HES was administered to the IG. The caregivers’ needs as well as knowledge of and attitudes to ARI were evaluated in both groups, before (M0) and two months after the HES (M1).

**Results:** At M0 the caregivers from both groups had ‘some or great need’ about all HES domains; at M1 the caregivers in the IG expressed ‘no or low need’, whereas the CG maintained ‘some or great need’ about all HES domains (0.011 ≤ *P* ≤ .047). Concerning caregivers’ knowledge of and attitudes to ARI, at M1 there was a higher frequency of caregivers with right answers in the IG than in the CG (IG =7.5 ± 1 versus CG =6.0 ± 2; *P* = .000). Those differences occurred in domain (e) nasal clearance techniques, revealing a higher percentage of caregivers who used correctly nasal irrigation (*P* = .000), nasal aspirators (0.000 ≤*P* ≤ .001) and nebulization (*P* = .000*)* in IG.

**Conclusion:** The HES met the caregivers’ needs regarding ARI and increased their knowledge and attitudes towards ARI, especially regarding nasal clearance techniques.

Key Messages

- Health education session specifically according to the caregivers’ expressed needs regarding acute respiratory infections in children.
- Improved caregivers’ knowledge and attitudes towards acute respiratory infections in children.
- Empowerment of caregivers regarding their child’s health.

## Introduction

Acute respiratory infections (ARI) are common in children under five years old [1]. There are several studied risk factors of ARI in small children, such as: mother’s educational background, lack of breastfeeding, overcrowding, incomplete immunisation, smoke exposure or attending day-care [1,2]. In fact, day-care attendance may increase the number of episodes of ARI by up to two or three times, once transmission is facilitated [1].

The increase in the frequency of a child’s illnesses causes much concern to caregivers and generally, they become uncertain regarding the management of their child’s ARI, feeling that it is safer to re-consult the general practitioner [3,4]. Re-consultation represents an opportunity cost and can increase the pressure on clinicians to prescribe antibiotics, increasing the socio-economic burden and affecting public health [1,5,6].

The concerns of parents can be minimized with the implementation of health education interventions in the community, addressing information, training and educating for caregivers, to achieve a correct management of children’s ARI [3,7,8,9].

This study aimed to design a health education session addressed to caregivers of children, and to evaluate its effects on caregivers’ needs, as well as on their knowledge and attitudes concerning ARI.

## Methods

### Setting

This study was conducted during winter (January to March 2015) in seven day-care centres in Porto, Portugal, including caregivers (parents and/or legal tutors) of children less than three years of age and excluding caregivers of children with preterm birth or chronic neuromuscular or respiratory diseases. Day-care centres are institutions that provide supervision and care of young children during the daytime, when parents are at work.

### Ethics

This study obtained ethical approval from the Ethics Committee of the School of Allied Health Technologies, Polytechnic Institute of Porto (CE_1744/2014) and is registered in ClinicalTrials.gov with the identifier: NCT02588963.

### Study design

A generalized model of developing, implementing and evaluating a community-based intervention was followed to design and evaluate the effects of a HES [10]. This model comprises five major steps throughout the planning and evaluating processes: [1] assessing needs, [2] setting goals and objectives, [3] developing an intervention, [4] implementing the intervention, and [5] evaluating the results [10].

#### Assessing needs

A pilot test was carried out to gather primary data from a group of 10 caregivers who answered the question ‘Which subjects would you like to understand better to manage the acute respiratory infections of your child?’ These answers were analysed using the Delphi’s method by an expert panel (three-blinded health professionals with at least five years of experience in the treatment of children with ARI), and grouped into five domains: (a) prevention of ARI, (b) first signs and symptoms of ARI, (c) worsening signs of ARI, (d) medication, (e) nasal clearance techniques (Table 1).

**Table 1.** Needs identified by 10 caregivers regarding acute respiratory infections in children and objectives defined by the panel of experts.

Domain	Caregivers’ expressed needs^a^	Objectives
(a) Prevention of ARI	84.3%	To inform caregivers of the general primary and secondary prevention measures.
(b) First signs and symptoms of ARI	72.5%	To inform caregivers about of the first signs of ARI. To teach caregivers how to manage the first signs and symptoms of ARI.
(c) Worsening signs of ARI	72.6%	To inform caregivers about of the worsening signs of ARI. To teach caregivers how to manage the worsening signs of ARI.
(d) Medication	70.6%	To inform caregivers about the importance of: letting the general practitioner decide when the child should take medication (especially antibiotics); complying with the dosages and frequencies of medication stablished by the GP; knowing that antibiotics are not effective against viral ARI.
(e) Nasal clearance techniques	74.6%	To teach caregivers how to use nasal irrigators, nasal aspirators and nebulization according to the child’s motor development.

#### Setting goals and objectives

The general goal of this community-based intervention was to increase caregivers’ knowledge, as well as to improve their attitudes towards ARI in children. To operationalize and achieve this goal, several objectives were defined according to the caregivers’ needs (Table 1).

#### Developing an intervention

Each domain of the HES was designed according to the caregivers’ needs and taking into account the existing evidence-based literature. The following subjects were included in the HES: (a) prevention of ARI: primary and secondary prevention measures; (b) first signs and symptoms of ARI: correct management of rhinorrhoea, cough and nasal congestion; (c) worsening signs of ARI: appropriate actions regarding fever, loss of appetite, dehydration, or signs of increased difficulty in breathing; (d) medication: decide with the child’s doctor when antibiotics should be taken, the appropriate dosage and frequency of medication, and when to stop; (e) nasal clearance techniques: demonstrative and shared practice about the appropriate way to use nasal irrigation according to the child’s age; remarks about the use of nasal aspirators and nebulization.

#### Implementing the intervention

The information concerning the HES was disseminated among the target population, scheduling meetings with the administrators of several day-care centres of Porto. After their approval, the caregivers were contacted and informed about the aims and procedures of this study, expressing their formal written consent according to the Declaration of Helsinki.

The HES had a mean duration of one hour to discuss the theoretical premises of the five domains, followed by 30 min of shared practice in nasal clearance techniques on paediatric models. A blinded respiratory physiotherapist conducted the HES with small groups of 10 to 15 caregivers at the day-care centre. At the end of the sessions, the participants received a small booklet with a summary of the information.

#### Evaluating the results (outcomes)

The effect of the HES on the caregivers’ needs regarding ARI, as well as on the caregivers’ knowledge and attitudes towards ARI, were analysed two months after the HES.

### Sample size and selection of study subjects

Pilot testing in a sample with 10 caregivers randomized into an intervention group (IG) who attended a HES and eight caregivers into a control group (CG) who were not allocated to any intervention showed mean differences regarding the caregivers’ knowledge after the HES (X_IG_ = 82.5% ± 3.83; X_CG_ = 67.7% ± 12.15). Sample size calculation revealed an effect size of 1.643 with a 95% power and at a 5% significance level, estimating a total sample size of 24 individuals (IG = 12; CG = 12).

Taking into account the large losses that generally occurs in longitudinal studies, we contacted 152 caregivers at seven day-care centres and 105 agreed to participate in the study (response rate = 69%). Seven children were excluded (six preterm births and one asthmatic) and, so a final sample of 98 caregivers was obtained. Then, the caregivers were randomly distributed by a blinded collaborator, according to a table of random numbers between 0 (CG; *n* = 51) and 1 (IG; *n* = 47), given by the statistical software.

The caregivers from the CG, as well as the day-care workers (educators and assistants) were invited to participate in an extra HES once the study ended.

### Measurements

The children’s caregivers were asked to fill in a registration form in order to collect sociodemographic characteristics, anthropometric data and risk profile history, as well as the Portuguese version of the Zung self-anxiety scale [11].

Caregivers’ needs were evaluated according to a ‘scale of needs’, which was built by the expert panel, including one question about each of the five domains to be answered on a Likert scale, varying from 1 (no need) to 5 (great need). The content validity of the ‘scale of needs’ was assured using the Delphi method and an excellent intra-rater reliability was found for each domain (0.71 < ICC < 1) according to Fleiss’ classification.

Caregivers’ knowledge of and attitudes to ARI were assessed by a written questionnaire of knowledge and attitudes towards ARI, which was designed by the expert panel, including two multiple-choice questions on each of the five domains of the HES. Content validity was assured and an excellent intra-rater reliability was obtained (ICC = 0.96) in a sample of six caregivers. The number of right answers in each of the domains, as well as the total number of right answers, was compared in both groups.

### Statistical analysis

All statistical analyses were carried out using IBM SPSS Statistics 22 for Windows 8 with a confidence interval of 95% (95%CI) (significance level of α = 0.05).

Descriptive statistics, respectively mean, standard deviation, median, interquartile range and frequencies were used. Inferential statistics was used, namely intraclass correlation coefficient (ICC) for intra-rater reliability, as well as Kendall tau-b correlation coefficient (ordinal variables), Mann–Whitney U-test (ordinal variables), chi-square test and Fisher’s exact test (dichotomous variables) for inter-group analysis.

## Results

### Participants

**Figure 1.**
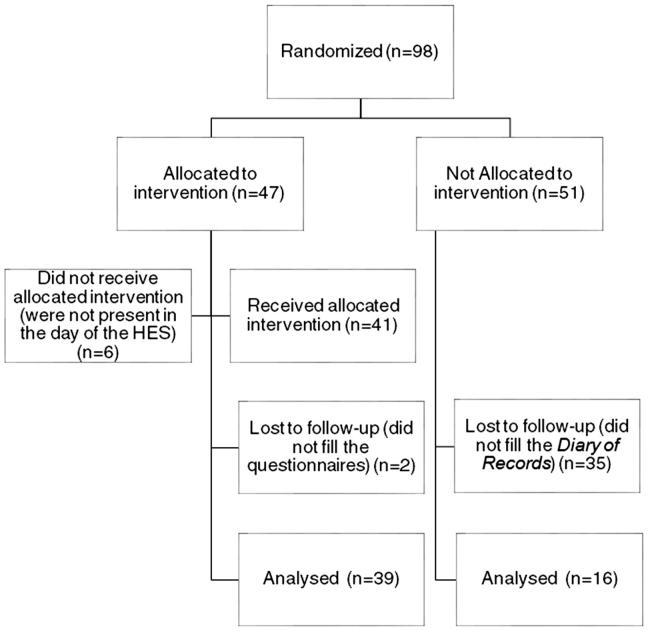
Participants diagram flow.

From a sample of 98 caregivers of children attending day-care centres, 47 were randomly distributed into an intervention group (IG) and 41 attended the HES (*n* = 41); the remaining 51 caregivers were randomized into a control group (CG) (*n* = 51) who were not allocated to any intervention (Figure 1).

Baseline demographic and clinical characteristics of children and caregivers for each group are summarized at Table 2.

**Table 2.** Risk factors for acute respiratory infections in children (intervention group versus control group).

Risk factors	Group	Continuous variables (X ± SD)	Dichotomousvariables (%)	*P* value (95%)
Mother				
Mother’s age at birth (years)	IG	31.6 ± 4.61	–	.794
	CG	31.3 ± 3.74	–	
Pregnancy duration (weeks)	IG	38.4 ± 1.92	–	.845
	CG	37.6 ± 1.89	–	
Breastfeeding (months)	IG	8.42 ± 6.41	–	.231
	CG	6.97 ± 4.32	–	
Mother’s anxiety (Zung’s score)	IG	32.6 ± 5.77	–	.624
	CG	31.8 ± 7.17	–	
Level of education (Higher)	IG	–	63.4	.270
	CG	–	78.3	
Household				
Household (>3)	IG	–	48.8	.192
	CG	–	30.4	
Siblings (yes)	IG	–	51.2	.124
	CG	–	30.4	
Parents’ respiratory diseases (yes)	IG	–	39.0	1.000
	CG	–	39.1	
House smoking (yes)	IG	–	22.0	.301
	CG	–	8.70	
Child				
Gender (male)	IG	–	53.7	.610
	CG	–	60.9	
Age (months)	IG	21.3 ± 9.34	–	.128
	CG	24.9 ± 8.20	–	
Weight at birth (kg)	IG	3.19 ± 0.45	–	.145
	CG	3.00 ± 0.42	–	
Day-care centre				
Room size (m^2^)	IG	27.60 ± 8.98	–	.734
	CG	28.39 ± 8.60	–	
Children per room (*n*)	IG	10.58 ± 4.11	–	.856
	CG	10.39 ± 3.35	–	

*P* ≤ .05 is considered to be significant.

IG, intervention group; CG, control group; X, mean; SD, standard deviation.

### Effect of the HES on the caregivers’ needs regarding ARI

Before the HES, most caregivers from both groups had some or great need for information on all of the HES domains. After the HES, there were significant differences between the groups regarding the needs expressed, the majority of the caregivers in the IG expressed no or low need for information while caregivers in the CG maintained some or great need for information about all of the HES domains. Detailed results are provided in Table 3.

**Table 3.** Caregivers’ needs regarding the domains of the Health Education Session before (M0) and two months after (M1) the Health Education Session (intervention group versus control group).

		(1) No need		(2) Low need		(3) Indifferent		(4) Some need		(5) Great need		*P* value (95%)	
		M0	M1	M0	M1	M0	M1	M0	M1	M0	M1	M0	M1
(a) Prevention of ARI	IG	3%	17.9%	3%	53.6%	6.1%	7.1%	54.5%	17.9%	33.3%	3.6%	.251	.014^a^
	CG	5.6%	20%	16.7%	20%	0%	10%	55.6%	50%	22.2%	0%		
(b) First signs and symptoms of ARI	IG	3%	17.9%	9.1%	64.3%	9.1%	7.1%	63.6%	7.1%	15.2%	3.6%	.553	.011^a^
	CG	5.6%	30%	16.7%	10%	16.7%	10%	38.9%	50%	22.2%	0%		
(c) Worsening signs of ARI	IG	3%	17.9%	9.1%	57.1%	9.1%	3.6%	54.5%	17.9%	24.2%	3.6%	.516	.012^a^
	CG	5.6%	10%	16.7%	40%	16.7%	10%	33.3%	40%	27.8%	0%		
(d) Medication	IG	3%	17.9%	12.1%	53.6%	12.1%	3.6%	48.5%	25%	24.2%	0%	.597	.018^a^
	CG	5.6%	20%	22.2%	20%	5.6%	20%	4.4%	40%	22.2%	0%		
(e) Nasal clearance techniques	IG	6.1%	21.4%	3%	39.3%	9.1%	10.7%	54.5%	25%	27.3%	3.6%	.348	.047^a^
	CG	5.6%	30%	22.2%	10%	11.1%	20%	33.3%	40’%	27.8%	0%		

^a^ *P* ≤ .05 is considered to be significant.

IG, intervention group; CG, control group; M0, before Health Education Session; M1, two months after Health Education Session.

### Effect of the HES on the caregivers’ knowledge of and attitudes to ARI

Before the HES, no significant differences between groups were observed in terms of the caregivers’ knowledge of and attitudes to ARI. After the HES, there was a higher frequency of caregivers with right answers in the IG than in the CG. When analysing caregivers’ answers in each domain, only (e) Nasal clearance techniques, showed significant differences between groups, revealing better results in the IG (Table 4).

**Table 4.** Frequency of caregivers with right answers in the questionnaire of knowledge and attitudes towards ARI in children (intervention group versus control group).

	M0 (%)					M1 (%)			
Number of right answers	0		1	2	*P* value (95%)	0	1	2	*P* value (95%)
Domain									
(a) Prevention of ARI	IG	5.6	61.1	33.3	.516	0	43.2	56.8	.094
	CG	18.2	50.0	31.8		6.7	60.0	33.3	
(b) First signs and symptoms of ARI	IG	11.1	63.9	25.0	1.000	5.4	51.4	43.2	.954
	CG	13.6	59.1	27.3		13.3	40.0	46.7	
(c) Worsening signs of ARI	IG	2.8	61.1	36.1	.262	0	45.9	54.1	.124
	CG	13.6	59.1	27.3		0	73.3	26.7	
(d) Medication	IG	5.6	61.1	33.3	.729	2.7	35.1	62.2	.581
	CG	9.1	50.0	40.9		6.7	40.0	53.3	
(e) Nasal clearance techniques	IG	55.6	44.4	0	.593	2.7	45.9	51.4	0^a^
	CG	63.6	36.4	0		66.7	33.3	0	
Caregivers’ total number of right answers (M ± IR)	IG	6.0 ± 2			.257	7.5 ± 1			0^a^
	CG	5.0 ± 2				6.0 ± 2			

^a^ *P* ≤ .05 is considered to be significant.

IG, intervention group; CG, control group; M0, before Health Education Session; M1, two months after Health Education Session; M, median; IR, interquartile range.

This led us to analyse further the results of the domain (e) Nasal clearance techniques, finding a significant higher percentage of caregivers in the IG who managed more correctly to use nasal irrigation, nasal aspirators and nebulization than the caregivers in the CG (Table 5).

**Table 5.** Frequency of caregivers’ answers concerning the domain ‘nasal clearance techniques’ in the written test (intervention group versus control group).

				M0 (%)	*P* value (95%)	M1 (%)	*P* value (95%)
Answers of caregivers concerning the domain (e) nasal clearance techniques	Nasal Aspirators	Does a slow and prolonged inspiration (Right answer)	IG	27.3	.500	84.2	0^a^
			CG	15.0		20.0	
		Does a fast and profound inspiration (Wrong answer)	IG	27.3	.375	7.9	.001^a^
			CG	40.0		53.3	
	Nasal Irrigation	Let the serum enter anostril and exit through the other (Right answer)	IG	15.2	.697	89.3	0^a^
			CG	10.0		30.0	
	Nebulization	After nebulization, lays down with the child (Wrong answer)	IG	51.5	1.000	2.6	0^a^
			CG	50.0		60.0	
		After nebulization, plays with the child (Right answer)	IG	12.1	.639	100	0^a^
			CG	5.0		13.3	

^a^ *P* ≤ .05 is considered to be significant.

IG, intervention group; CG, control group; M0, before Health Education Session; M1, two months after Health Education Session.

## Discussion

### Main findings

This study found that, two months after attending the HES, most of the caregivers in the IG expressed ‘no or low need’ for information concerning ARI in children, while the CG maintained ‘some or great need’ for information. This means that the HES met the parents’ needs concerning the management of respiratory infections. The HES have also positively influenced caregivers’ knowledge and attitudes to ARI, which persisted two months after the HES.

### Strengths and limitations

The major strength of this study was the conception of the HES according to the caregivers’ expressed needs, which allowed us to know what the caregivers’ concerns about ARI were and fulfil them in the HES. Further, it was assured that children from both groups were exposed to the same influence of the risk factors of ARI, allowing us to assume that the results obtained in the IG regarding caregivers’ knowledge and attitudes to ARI were due to the HES.

Nevertheless, this study faced some limitations. We were not able to measure if the caregivers actually change their behaviour or if they performed the nasal clearance techniques appropriately, only that they increased their knowledge and had a better attitude towards it. There were a high number of participants who were lost to follow-up throughout two months, especially in the CG. The positive short-term effects of the HES on the awareness and motivation of the participants, which might be independent of the contents of the HES, can be the reason why there were a smaller number of losses in the IG. Furthermore, the small sample size compromises the external validity of our findings.

### Interpretation of the study results in relation to existing literature

The caregivers’ needs were grouped into five general domains: prevention, signs and symptoms, worsening, medication, and nasal clearance techniques. More than three quarters of the caregivers expressed the need to know more about domain (a) prevention of ARI. Prevention is a very important concern due to the high incidence of ARI in children, especially among those attending day-care centres. Still, some researchers showed that parents consider some of the preventive measures difficult to implement in children, especially regarding social distancing behaviours or avoiding day-care when the child is ill [1,12]. Day-care centres constitute an environment highly favourable for the transmission of common respiratory pathogens, given the increased exposure of children to pathogens, their close contact with each other and the typical limitations of their own hygiene due to the age factor [1,13,14,15]. Therefore, it is fundamental to increase the awareness of caregivers to the importance of the preventive measures, since they are effective and can decrease the risk of ARI [16].

Concerning domain (b) first signs and symptoms of ARI, most caregivers expressed ‘some or great need’ for information before the HES. Similarly, regarding domain (c) worsening signs of ARI, more than a half of the caregivers expressed ‘some or great need’ for information. Previous studies have indicated that parents need information to help them understand and manage the illness of their child, including signs of serious illness, how to care for the child, what is normal and how to prevent or reduce future episodes [4]. In fact, parents often feel uncertain about identifying and interpreting signs and symptoms of ARI [3–5], causing them to seek often for their general practitioner, thus increasing the burden of the disease [4,17]. Some studies found that providing parents with proper health information prior to their child becoming ill resulted in lower rates of consulting for respiratory infections [18–20].

In domain (d) medication many caregivers showed ‘some or great need’ for information. Many parents have misconceptions about antibiotic resistance, believing that antibiotics can decrease the duration of ARI symptoms as well as the incidence of ARI complications [3,8,9]. Vodicka and colleagues reported in their systematic review that interventions directed towards parents and/or clinicians can reduce rates of antibiotic prescribing [21].

Nevertheless, it is important to make parents aware that they can manage their child’s ARI with other methods besides medication, since there is insufficient evidence of an effective pharmacological treatment of URTI in children [12].

Therefore, it is fundamental to explore non-pharmacological methods to manage URTI, as expressed by half of the caregivers who had ‘some or great need’ for information about domain (c) nasal clearance techniques. In this study, most of the caregivers reported that they use nasal clearance techniques when their child has rhinorrhoea. However, according to their answers before the HES, it was clear that they did not perform them correctly. Indeed, most general practitioners prescribe nasal saline irrigation due to its benefits in infants and children, relieving symptoms of URTI, such as rhinorrhoea or nasal congestion, as well as reducing the use of medication [22,23]. However, there is little agreement regarding the dosage and the tonicity of saline solutions, as well as the frequency of application and the positioning of the child, causing parents’ insecurity [24,25]. In our study the caregivers in the IG had improved their knowledge of and attitude to nasal clearance techniques, which may also be due to the fact that, during the HES, the caregivers were able to practice these techniques on paediatric models. Experience seems to be an important key factor which parents reported increased their self-efficacy and thus reduce their need to consult or re-consult the general practitioner [4].

### Implications for clinical practice

Health education of caregivers is vital to an effective prevention or management of ARI in children. Community-based interventions are needed in at-risk populations in order to meet the caregivers’ needs and concerns regarding ARI. This may decrease the number of general practice consultations and re-consultations as well as the pressure on the GP to prescribe antibiotics, improving public health.

## Conclusion

The Health Education Session met the caregivers’ needs regarding ARI and increased the caregivers’ knowledge of and attitudes to ARI, especially with regard to nasal clearance techniques.
